# The Mst1 Kinase Is Required for Follicular B Cell Homing and B-1 B Cell Development

**DOI:** 10.3389/fimmu.2018.02393

**Published:** 2018-10-17

**Authors:** Faisal Alsufyani, Hamid Mattoo, Dawang Zhou, Annaiah Cariappa, Denille Van Buren, Hanno Hock, Joseph Avruch, Shiv Pillai

**Affiliations:** ^1^Ragon Institute of MGH, MIT and Harvard, Massachusetts General Hospital, Harvard Medical School, Boston, MA, United States; ^2^Department of Molecular Biology, Massachusetts General Hospital, Harvard Medical School, Boston, MA, United States; ^3^Center for Cancer Research, Massachusetts General Hospital, Harvard Medical School, Boston, MA, United States

**Keywords:** B-1 B cell, kinase, spleen, MST1 (mammalian sterile 20-like kinase 1), marginal zone (MZ) B cells, follicular 1 and 2 B-cells

## Abstract

The Mst1 and 2 cytosolic serine/threonine protein kinases are the mammalian orthologs of the Drosophila Hippo protein. Mst1 has been shown previously to participate in T-cell and B-cell trafficking and the migration of lymphocytes into secondary lymphoid organs in a cell intrinsic manner. We show here that the absence of Mst1 alone only modestly impacts B cell homing to lymph nodes. The absence of both Mst1 and 2 in hematopoietic cells results in relatively normal B cell development in the bone marrow and does not impact migration of immature B cells to the spleen. However, follicular B cells lacking both Mst1 and Mst2 mature in the splenic white pulp but are unable to recirculate to lymph nodes or to the bone marrow. These cells also cannot traffic efficiently to the splenic red pulp. The inability of late transitional and follicular B cells lacking Mst 1 and 2 to migrate to the red pulp explains their failure to differentiate into marginal zone B cell precursors and marginal zone B cells. Mst1 and Mst2 are therefore required for follicular B cells to acquire the ability to recirculate and also to migrate to the splenic red pulp in order to generate marginal zone B cells. In addition B-1 a B cell development is defective in the absence of Mst1.

## Introduction

Immature B cell migrate from the bone marrow to the spleen as newly-formed transitional type 1 B cells and they then enter the splenic follicle where they become type 2 transitional B cells, acquire the expression of CD23 and IgD, mature into follicular B cells and gain the functional ability to recirculate (1–3). Recirculation is a key biological function of follicular B cells that allows them to home to the lymph nodes and the bone marrow in order to be engaged by cognate antigens and acquire T cell help (3–5). The precise molecular requirements for the acquisition of the ability of follicular B cells to recirculate have not been established.

Some transitional type 2 B cells in the splenic white pulp and possibly IgM and IgD expressing follicular B cells (FO-II B cells) migrate to the red pulp of the spleen, contact Notch ligands and are induced to differentiate into marginal zone B cell precursors (MZP B cells) and marginal zone (MZ) B cells (6–10). We have argued that B cells that recognize self-antigens relatively weakly may be selected to differentiate into MZ B cells (3, 11, 12). Many studies have revealed the need for intact splenic architecture to generate marginal zone B cells, and for integrins and molecules linked to integrin signaling to retain B cells in the marginal zone (13–18).

The mammalian Mst 1 and 2 protein kinases are orthologs of the Hippo protein in *Drosophila* which plays a crucial role in controlling organ size by its ability to regulate cellular proliferation and apoptosis (19, 20). In mammalian cells Mst 1/2 phosphorylate the downstream kinase LATS1 that phosphorylates and inactivates Yap which is retained in the cytoplasm when phosphorylated (21–23). The absence of Hippo pathway activation leads to the translocation of Yap to the nucleus where it binds to different transcription factors that typically induce the expression of genes responsible for cell growth and survival (24–28).

Mst1 has been shown to be activated in lymphocytes downstream of chemokine receptor activation, and in this context the Mst kinases function independently of LATS and Yap, but activate the NDR1 and NDR2 kinases that are homologs of LATS (29). The Mst/Ndr pathway has been linked to actin polarization, lymphocyte motility and the regulation of lymphocyte migration and homing to secondary lymphoid organs in a cell intrinsic manner. Lymphopenia has been observed in the absence of Mst1, but although marginal zone B cell numbers have been shown to reduce in the absence of this kinase, reported reductions in follicular B cells were relatively modest (30).

We report here that in the absence of both Mst1 and Mst2, B cells develop normally in the bone marrow, emigrate to the spleen and develop into cells with a follicular B cell phenotype. However there is a near total absence of B cell seeding of lymph nodes and recirculation to the bone marrow. In addition follicular B cells in the spleen are constrained to the white pulp and do not reach the red pulp, providing an explanation for the absence of marginal zone B cells. These data suggest that Mst1 and 2 are required for follicular B cells to acquire the ability to recirculate, a key functional attribute that defines this subset of lymphocytes. In addition, in the absence of Mst1, B-1a B cell development is significantly compromised.

## Results

### Striking reduction of B cells in lymph nodes in the absence of both Mst1 and Mst2

**Figure 1:**
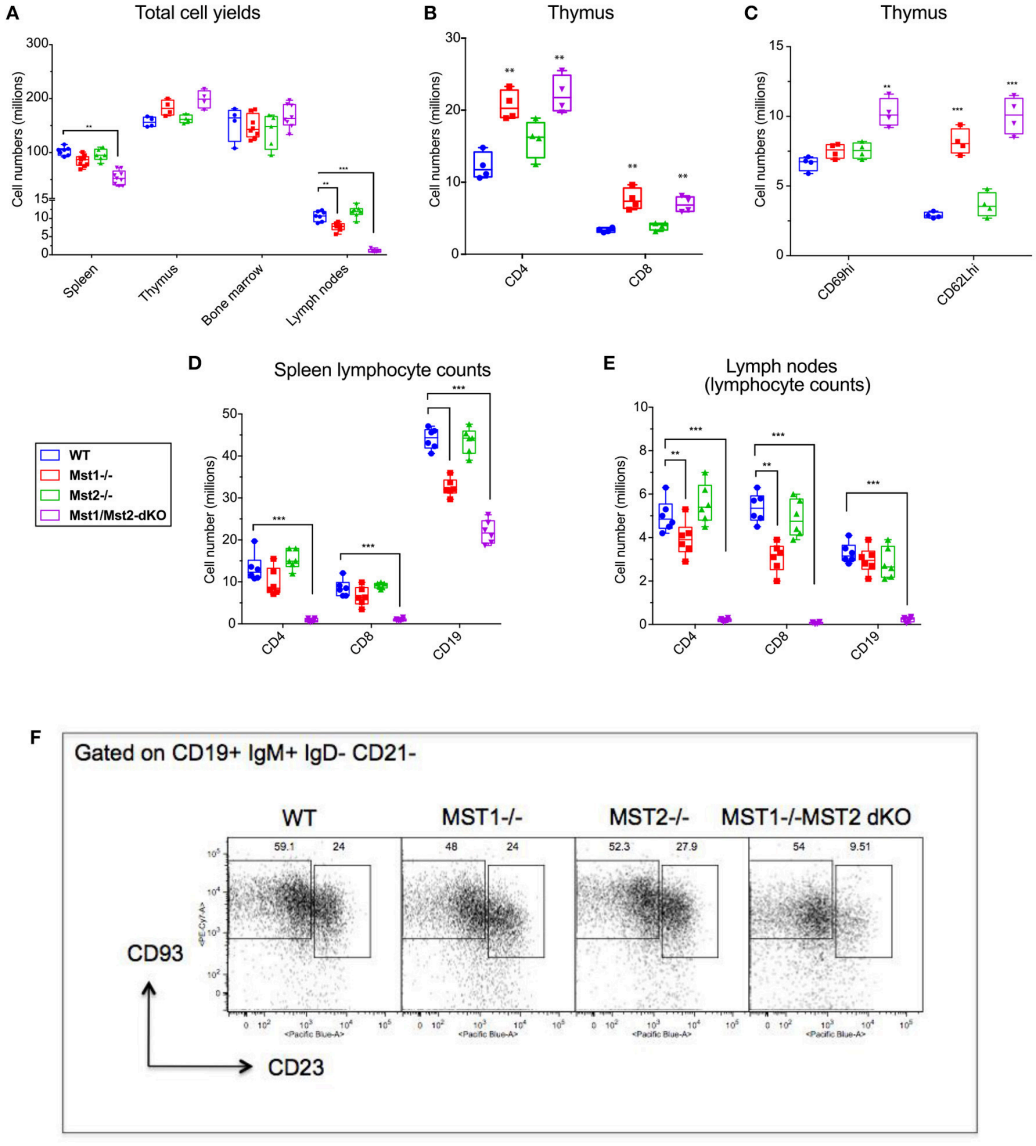
Lymphocyte counts from primary and secondary lymphoid organs of Mst1^−/−^ and Mst1/Mst2-dKO mice: **(A)** Total cell yields from Spleen, LN, bone marrow, and Thymus harvested from Mst1^−/−^, Mst2^−/−^, Mst1/Mst2-Dko, and WT controls at 8 weeks of age. (*n* = 6–7, error bars depict mean ± SD). **(B)** Total CD4-SP and CD8-SP T cell counts from thymus harvested from Mst1^−/−^, Mst2^−/−^, Mst1/Mst2-dKO and WT controls at 8 weeks of age. (*n* = 4, error bars depict mean ± SD). **(C)** Total immature and mature CD4-SP T cell counts from thymus harvested from Mst1^−/−^, Mst2^−/−^, Mst1/Mst2-dKO, and WT controls at 8 weeks of age. (*n* = 4, error bars depict mean ± SD). **(D)** Total CD4+ and CD8+ T cell and CD19+ B cell counts from spleens harvested from Mst1^−/−^, Mst2^−/−^, Mst1/Mst2-dKO, and WT controls at 8 weeks of age. (*n* = 5–6, error bars depict mean ± SD). **(E)** Total CD4+ and CD8+ T cell and CD19+ B cell counts from pooled popliteal, inguinal, and axillary lymph nodes harvested from Mst1^−/−^, Mst2^−/−^, Mst1/Mst2-dKO, and WT controls at 8 weeks of age. (*n* = 5–6, error bars depict mean ± SD). **(F)** Flow cytometry analysis of T1 and T2 transitional B cells in the spleen. **p < 0.01, ***p < 0.001.

In order to assess the individual contributions of Mst1 and Mst2 in hematopoiesis and to address their functional redundancy, we analyzed primary and secondary lymphoid organs from *Mst1*^−/−^, *Mst2*^−/−^ as well as *Vav-Cre Mst1*^−/−^*Mst2*^*F*/*F*^ [Mst1/Mst2 double knockout (DKO)] mice for different lymphoid compartments. We initially quantitated total lymphocyte numbers in the spleen, bone marrow, thymus and lymph nodes in wild type littermate control mice, *Mst1*^−/−^ mice, *Mst2*^−/−^ and *Mst1/Mst2-dKO* mice (Figure 1A and Supplementary Figure 1). No change in overall bone marrow and thymic lymphocyte numbers was observed in *Mst1*^−/−^*, Mst2*^−/−^, or *Mst1/Mst2-dKO* mice, but there was a reduction in splenic cell yields in *Mst1* ^−/−^ mice and more so in *Mst1/Mst2-dKO* mice (Figure 1A). These differences in cell yields were more pronounced in lymph nodes harvested from these mice. Also, there was an increase in thymic single positive CD4^+^ (CD4 SP) and CD8^+^ SP T cells in mice lacking *Mst1* and both *Mst1* and *Mst2* (Figure 1B) consistent with what has been described previously (31). Single positive CD4+ and CD8+ thymocytes increase the cell surface abundance of CD62L during their maturation while decreasing the expression of CD69. There is an accumulation of CD62Lhi cells in the CD4 SP as well as CD8 SP compartment that accounts for the overall increased cell counts (Figure 1C) and is likely to result from failed egress of SP thymocytes into the periphery. Total lymphocyte numbers in the spleen and lymph node were only modestly reduced in *Mst1*^−/−^ mice, but the total numbers of splenic lymphocytes were reduced to about 60% of the WT levels in mice lacking both Mst1 and Mst2; in contrast there was a striking reduction of total lymphocytes in lymph nodes to < 10% of normal levels in mice lacking both these kinases (Figure 1A). While in the absence of both Mst1 and Mst2 splenic T cell numbers were strikingly reduced, the reduction of total splenic B cell numbers was more modest (Figure 1D). In contrast, the absence of both Mst1 and Mst2 resulted in a striking reduction of both T and B cells in lymph nodes (Figure 1E). We directly examined transitional T1 (IgM+CD93+CD23−) and T2 (IgM+CD93+CD23+) B cells in the spleen and noted that T2 cells were diminished in the absence of both Mst1 and Mst2 (Figure 1F).

We also checked the peripheral blood for T and B cells, and noted a marked reduction of these circulating cells, consistent with previous results [(30, 31), Supplementary Figure 2]. The recirculation of lymphocytes is an acquired ability to migrate from one secondary lymphoid organ to the next and is distinct from specific homing events such as the emigration of immature B cells from the bone marrow to the spleen. Newly formed T1 B cell numbers in the spleen are not diminished in the absence of Mst1 and Mst2, and as shown later, bone marrow B cell production is grossly normal in the absence of these kinases. These results suggest that the absence of both Mst1 and Mst2 results in a striking loss of the ability of lymphocytes to recirculate. While this ability is acquired in the thymus by recently generated single positive T cells, the corresponding acquisition of recirculating ability by B cells occurs in the spleen. The loss of the ability to recirculate may theoretically reflect a loss of entry of T1 B cells from the red pulp into the splenic follicle, or a loss of B cell maturation from the T1 stage into the follicular T2 and mature follicular stages or possibly reflect the apoptotic death of recently generated T2 and follicular B cells in the white pulp. However, it has been described that Btk signaling may be defective in B cells in the absence of Mst1 (32) and our own data on BCR induced calcium flux in the absence of Mst1 suggests that BCR signaling is defective in the absence of these kinases (Supplementary Figure 3); this is consistent with the absence of mature follicular B cell maturation in the absence of Btk signaling (3). Since T1 B cells are short-lived (2) they may fail to accumulate in the red pulp in the absence of follicular B cell maturation.

### Altered peripheral development of B cells in mice lacking Mst1 and Mst2

**Figure 2:**
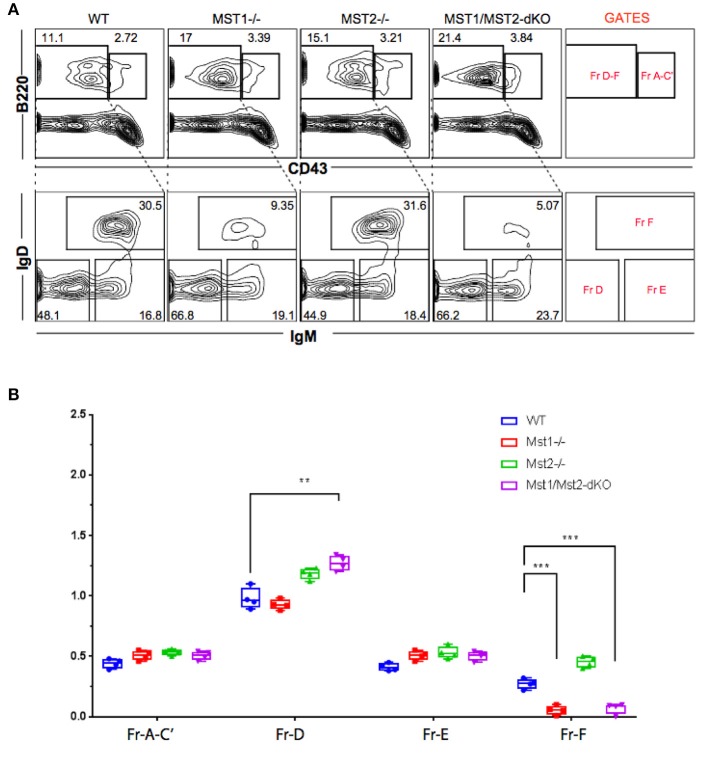
Reduced frequency of perisinusoidal B cells in Mst1^−/−^ and Mst1/Mst2-dKO mice. **(A)** Flow cytometric analyses depicting frequencies of different stages of early developing B cells in Bone Marrow of Mst1^−/−^, Mst2^−/−^, Mst1/Mst2-dKO mice, and WT controls. These flow plots are representative of 5 mice analyzed. The lower panel shows a reduction in the frequency of perisinusoidal B cells (Fraction F) in BM of Mst1^−/−^ and Mst1/Mst2-dKO. **(B)** Absolute cell counts of BM B cell fractions in Mst1^−/−^, Mst2^−/−^, Mst1/Mst2-dKO mice and WT controls. (*n* = 5, error bars depict mean ± SD, ***p < 0.001, **p < 0.05).

**Figure 3:**
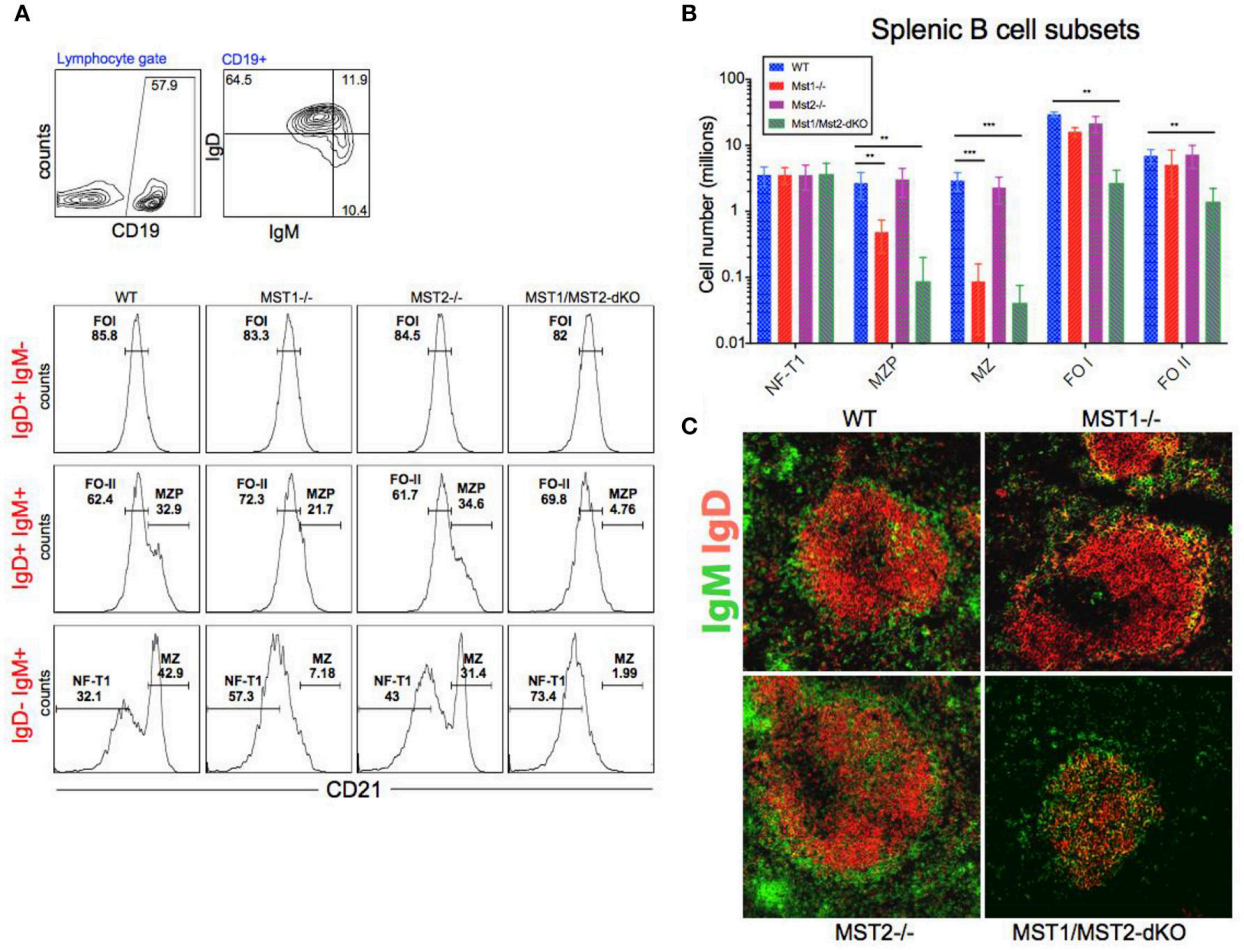
Depletion of Mst1 alone or both Mst1/Mst2 affects the development of MZ B cells as well as FO B cells in the periphery. **(A)** Flow cytometric analysis shows accumulation of IgD^−^IgM^+i^CD21^low^ NF-T1 B cells in Mst1/Mst2-dKO mice. There is a striking loss of IgD^−^IgM^+^CD21^+^ MZ B cells in Mst1^−/−^ and Mst1/Mst2-dKO mice. These flow plots are representative of 10 mice analyzed. (Gating strategy: CD19^+^ IgD^−^IgM^+^CD21^low^ NF-T1 B cell, CD19^+^IgD^+^IgM^+^CD21^hi^ MZP B cell, IgD^−^ IgM^+^ CD21^+^ MZ B cell, CD19^+^IgD^+^IgM^−^CD21^mid^ FO I B cell, and CD19^+^IgD^+^IgM^+^CD21^mid^ FO II B cell). **(B)** Absolute counts of different B cell fractions in spleens of Mst1^−/−^ (red), Mst2^−/−^ (magenta), Mst1/Mst2-dKO (purple) mice and WT controls (blue). (*n* = 7, error bars depict mean ± SD, ***p < 0.001, **p < 0.05). **(C)** Immunofluorescence on spleen sections shows an absence of the MZ B cell compartment in Mst1^−/−^ and Mst1/Mst2-dKO mice compared to the WT controls. Mst2^−/−^ mice have an intact MZ compartment.

Bone marrow B cell development is grossly normal in the absence of Mst1 or Mst2 individually as depicted by normal distribution of early B cells into Hardy's fractions A-C', D, and E (Figures 2A,B). Although the homing of follicular B cells to lymph nodes is not significantly impacted by the loss of Mst1 alone, bone marrow recirculating B cells (Hardy's Fraction F) are severely reduced in both *Mst1*^−/−^ mice as well as in *Mst1Mst2-dKO* mice (Figures 2A,B). In *Mst1Mst2-dKO* mice, although the spleen is strikingly bereft of T cells, IgD^−^IgM^+i^CD21^low^ newly formed/T1 B cells are abundant but they lack IgD^−^ IgM^+^ CD21^+^ Marginal zone (MZ) B cells and IgD^+^IgM^+^CD21^+^ MZ B cell precursors (MZP) (Figures 3A,B). The reductions in MZ B cell frequency and numbers are quite drastic as can also be seen by the absence of a ring of IgM^+^ MZ B cells around the follicle (demarcated by IgD staining) (Figure 3C). CD93^+^CD23^+^ Transitional type 2 (T2) B cells and CD19^+^IgD^+^IgM^−^CD21^mid^ follicular type1 (FO-I) and CD19^+^IgD^+^IgM^+^CD21^mid^ follicular type 2 (FO-II) B cells were all found in the spleen though in reduced numbers (Figures 3B, 1F), while CD93^+^CD23^−^ transitional type 1 (T1) B cells numbers were normal. Since MZ B cells showed the most striking reduction in Mst1^−/−^ and Mst1/Mst2-dKO mice, we asked whether the lack of MZ B cells was linked in some way to a defective migration event.

### Defective follicular B cell migration to the splenic red pulp in mice lacking Mst1 and Mst2

**Figure 4:**
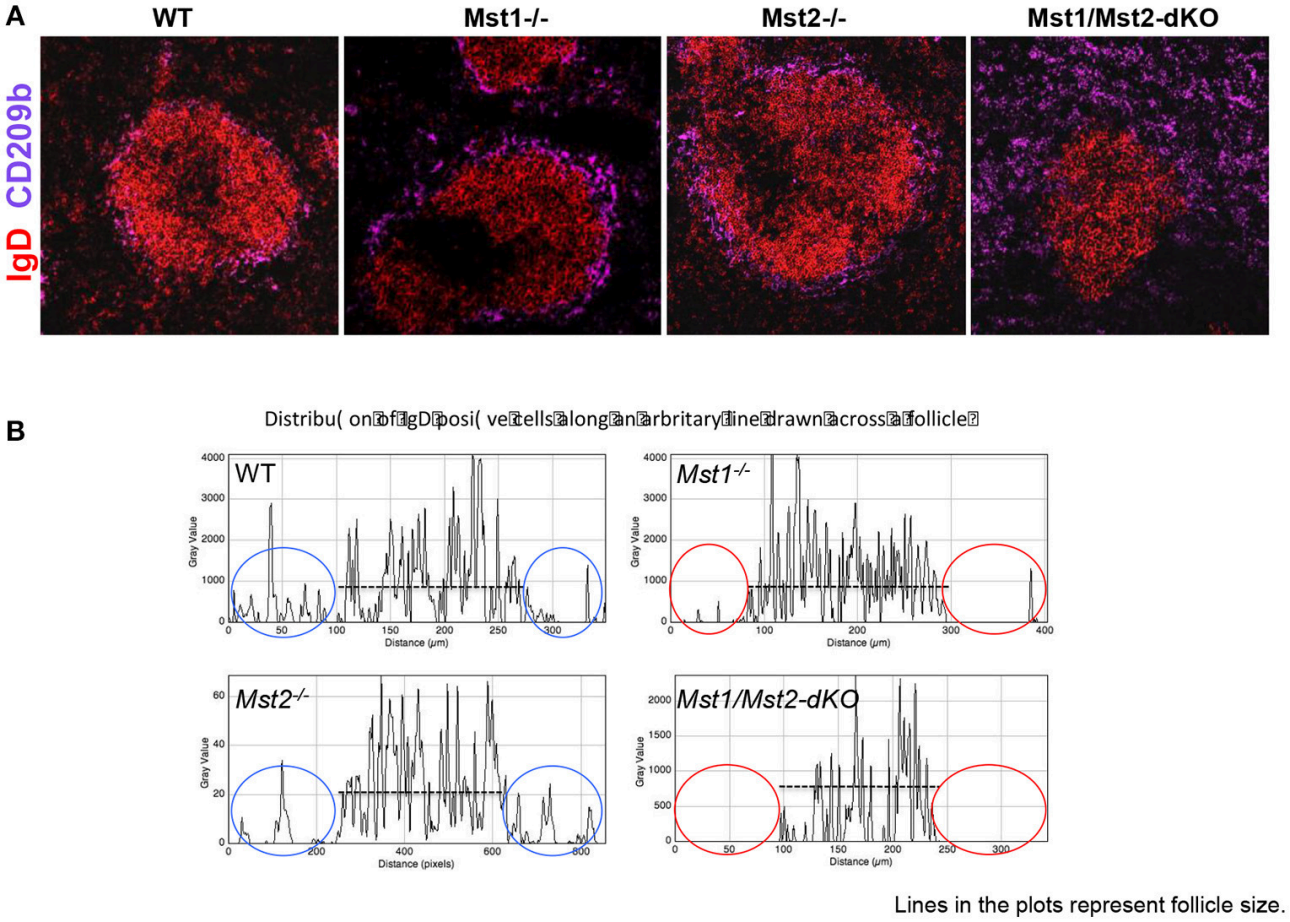
Disrupted architecture of the marginal compartment in Mst1/Mst2-dKO mice. **(A)** Immunofluorescence on spleen sections shows an intact MZ macrophage ring in Mst1^−/−^ and Mst2^−/−^ mice but a disrupted MZ macrophage compartment in Mst1/Mst2-dKO mice. IgD is in red and CD209b is in magenta. **(B)** Histogram plots generated on Zen software from spleen sections of Mst1^−/−^, Mst2^−/−^, Mst1/Mst2-dKO mice, and WT controls showing the distribution of IgD^+^ cells along a line drawn across the follicle extending into the red pulp. The red ovals highlight the lack of IgD+ B cells outside the follicles in Mst1^−/−^ and Mst1/Mst2-dKO mice. (Data are representative of spleens sectioned and analyzed from 3 mice of each genotype).

Defects in splenic architecture can compromise marginal zone B cell development. We examined the marginal compartment of splenic follicles using antibodies that specifically detect marginal zone macrophages. Marginal zone architecture was grossly normal in the absence of Mst1 and Mst2 individually (Figure 4A) as can be visualized by a ring of CD209b^+^ MZ macrophages comparable to that of the WT littermate controls. However, in Mst1/Mst2-dKO mice, there is a complete loss of architecture and CD209b^+^ MZ macrophages are scattered across the spleen without any proper organization.

Although migration of newly formed B cells from the bone marrow to the spleen is not defective in the absence of Mst1 and 2, and B cells mature in the splenic follicle, we considered the possibility that CD23 and IgD expressing transitional type 2 and follicular B cells must acquire the ability to migrate to the red pulp and interact with Notch ligands in order to acquire a marginal zone B cell fate or to be maintained as marginal zone B cells. We therefore examined the relative localization of B cells in and around the B cell follicle in wild type, *Mst1*^−/−^*, Mst2*^−/−^, and *Mst1Mst2-dKO* mice. As seen in Figure 4B, in the absence of Mst1 (or in mice lacking both Mst1 and Mst2 in hematopoietic cells) B cells fail to move away from the follicle into the red pulp. These data argue that the reason for defective MZP and MZ B cell development in the absence of Mst1 maybe the failure of Mst1 deficient B cells to access the red pulp.

**Figure 5:**
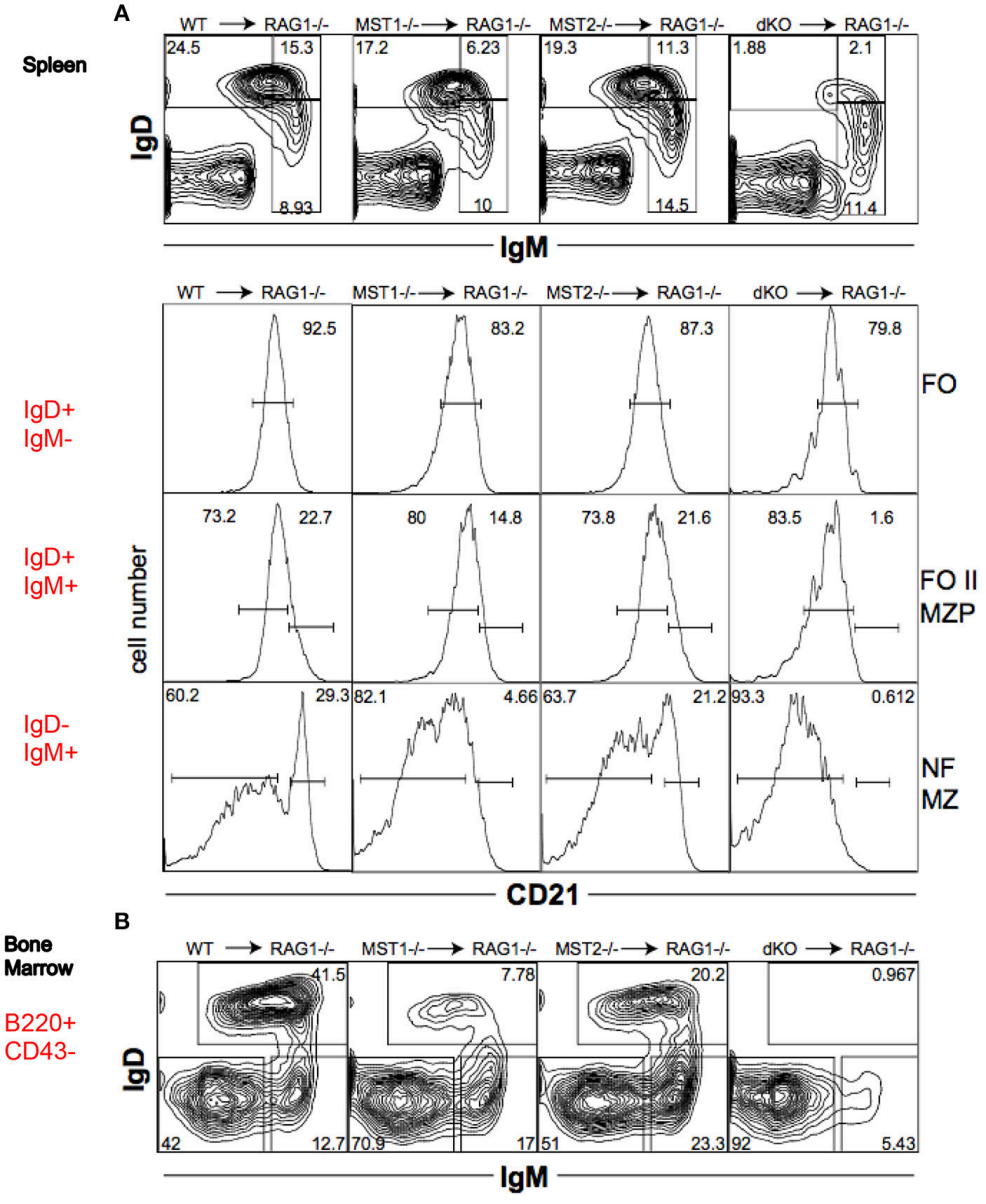
Mst1 and Mst2 are important for normal B cell development in a cell intrinsic manner. Rag-1^−/−^ mice were reconstituted with WT or mutant BM. Spleen and BM cells were analyzed 7 weeks later in the reconstituted mice. (Data are representative of 3 reconstituted mice for each group). **(A)** Reconstitution of different B cell sub-fractions in the reconstitute Rag1^−/−^ mice. Both IgD^+^IgM^+^CD21^+^ MZP and IgD^−^IgM^+^CD21^+^ MZ fractions are impacted. **(B)** Reconstitution of the different B cell fractions in the bone marrow of recipient Rag1^−/−^ mice. B220^+^CD43^−^IgD^+^IgM^+^ fraction F is depleted in Mst1^−/−^ and Mst1/Mst2-dKO mice.

To determine whether the alteration in peripheral B cell populations reflected a cell-intrinsic defect in function of Mst1 and Mst2 kinases, we reconstituted *Rag-1*^−/−^ mice with hematopoietic stem cells isolated from bone marrows of WT, *Mst1*^−/−^, *Mst2*^−/−^ and *Mst1/Mst2-dKO* mice. *Mst1*^−/−^ and *Mst1/Mst2-dKO* lymphocytes in reconstituted mice exhibited a defect in MZ B cell (IgD^−^IgM^+^CD21^+^) and MZP B cell (IgD^+^IgM^+^CD21^+^) development (Figure 5A) and a reduction of recirculating Hardy Fraction F BM B cells (B220^+^CD43^−^IgM^+^IgD^+^) (Figure 5B), which lends support to the hypothesis that Mst1 knockouts could adversely affect MZ development and recirculation of B cells in a lymphocyte-intrinsic manner.

### Reduced B-1a B cell numbers in Mst1^−/−^ and Mst1/Mst2-dKO mice

**Figure 6:**
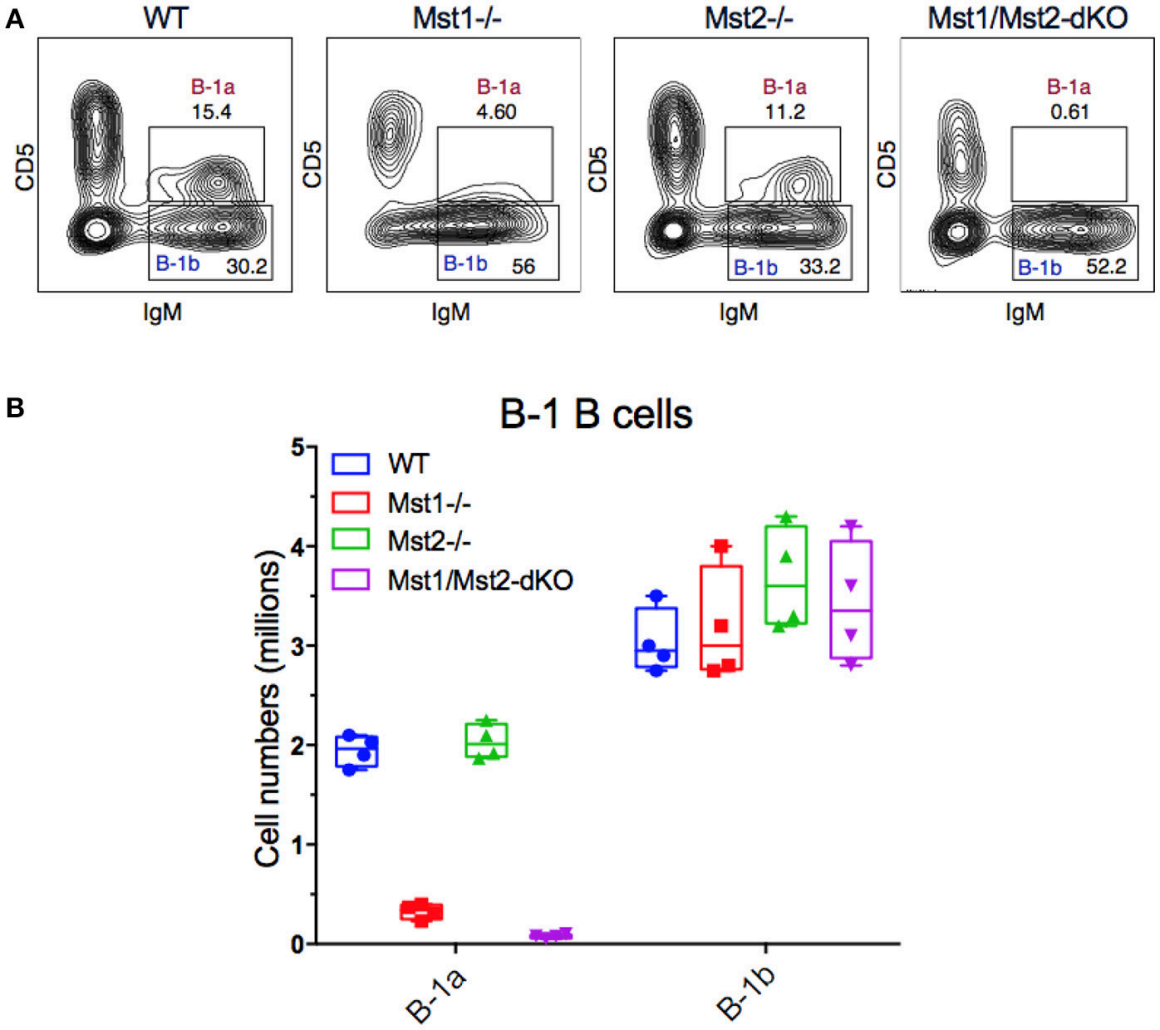
Loss of the B-1a B cell compartment in Mst1^−/−^ and Mst1/Mst2-dKO mice. **(A)** Flow cytometric analysis show a reduction of the CD5^+^IgM^+^ B-1a B cell compartment in the peritoneal cavity of Mst1^−/−^ and Mst1/Mst2-dKO mice. The B-1b B cell compartment in Mst2^−/−^ is intact. (Data are representative of 4 mice analyzed for each group). **(B)** Absolute cell counts of B-1a and B-1b B cell subsets in the peritoneal cavity of WT and mutant mice. (Box plots represent mean ± SE, *n* = 4).

Since B-1 cells have to migrate from the fetal liver to the peritoneal cavity, and strong BCR signaling is also required for B-1a B cell development, we examined B-1 cells in the peritoneum in mice lacking Mst1 and/or Mst2 kinases. The absence of Mst1 alone markedly reduced CD5^+^ B-1a B cell numbers but CD5^−^ B-1b B cells were minimally affected (Figures 6A,B). In the absence of both Mst1 and Mst2 a profound loss of B-1a B cells was observed but B-1b cell numbers were mostly unaffected.

## Discussion

It has been previously established that Mst1 and Mst2 participate in chemokine and S1P mediated cell migration events in lymphocytes (33). The absence of Mst1 and Mst2 has been shown to induce severe lymphopenia (30, 31) and disruption of Mst1 results in a cell-intrinsic defect of thymic egress of T cells presumably because of a defective response to chemokines. It was also suggested that in the absence of Mst1 there may be an impairment of integrin-dependent arrest of lymphocytes on HEVs.

By deleting both Mst1 and Mst2 in hematopoietic cells we have established that B cell emigration from the bone marrow to the spleen is well preserved but follicular B cells fail to acquire the ability to recirculate. While Mst1 alone plays a major role in B cell recirculation to the bone marrow and B cell migration to the red pulp, it is only in the absence of both Mst1 and Mst2 in hematopoietic cells that a profound defect in lymph node seeding is observed.

These data help establish that during B cell development, entry into the white pulp and acquisition of the follicular phenotype precedes the acquisition of the ability to recirculate and that this latter ability of follicular B cells requires Mst1 and Mst2. We also provide data to suggest that one of the mechanisms by which marginal zone B cell development occurs is the acquisition by transitional type 2 and follicular B cells of the ability to migrate to the splenic red pulp, and this migration event is dependent on the Mst1 kinase.

While single positive T cells acquire the ability to recirculate in the thymus itself, the major subset of B-2 cells, follicular B cells, matures mainly in the spleen and it is the spleen that these cells acquire the ability to recirculate. Although we and others have shown some B cell maturation occurs in the bone marrow itself (34, 35), in the absence of recirculation, it is evident that maturation in the bone marrow is minimal. While our results support a cell-intrinsic role for Mst1 in B cell development they leave open a cell-extrinsic role for this kinase as well. Transfers of wild type hematopoietic stem cells into Mst1 and Mst1/2 knockout mice would have to be undertaken to properly address that possibility.

## Materials and methods

### Mice

Mst1^−/−^, Mst2^−/−^ and Vav-Cre Mst1^−/−^Mst2F/F mice were maintained at the animal facility at Massachusetts General Hospital. C57 BL/6 (WT) and Rag1^−/−^ mice purchased from the Jackson Laboratory. Rag1^−/−^ mice transferred with cells from each strain (Bone marrow chimeras) were maintained for 8 weeks at the animal facility and were given acidified water. All animal procedures were approved by the sub-committees on research animal care at Massachusetts General Hospital.

### Antibodies, cell staining and flow cytometry

Following antibodies were used for flow cytometry: anti-mouse CD4-PE, anti-mouse CD8-FITC, anti-mouse CD25-APC, anti-mouse CD44-APC Cy7, anti-mouse CD90.2-PECy7, anti-mouse CD19-Pacific Blue, anti-mouse B220-PE, anti-mouse CD43-PE-Texas Red, anti-mouse IgM-APC, anti-mouse IgD-FITC, anti-mouse CD21-PE, anti-mouse CD23-Pacific Blue, anti-mouse CD93-PE Cy7 (AA4.1). All antibodies were purchased from Biolegend and BD Biosciences.

Single cell suspensions were made from spleens, lymph nodes (LNs) and bone marrow and RBCs were lysed (except in LNs) with 2 ml ACK lysing buffer (Lonza). The lysis buffer was neutralized by adding 10 ml HBSS and 0.2% BSA (Sigma). Before staining, 1 × 10^6^ cells were blocked with 2.5 μg of 2.4G2 (Biolegend) for 20 min on ice. Surface staining was performed using appropriate dilutions of antibodies in 96 well round-bottom plates in a volume of 100 μl of cell staining buffer (Biolegend, CA) for 30 min in the dark at 4°C or on ice. Flow cytometry was performed as on 4-laser BD LSR-II (BD Biosciences). Unstained cells were used to set voltage and single color positive controls were used for electronic compensation. Annexin V staining was performed in annexin binding buffer using Annexin-Pacific Blue or Annexin-Alexa Fluor 647 (Biolegend, CA) to score/gate-out apoptotic cells. Processed samples were analyzed on Flow Jo 9.3.1 (Tree Star, Inc OR).

### Immunofluorescence microscopy and image analysis

Spleens were fixed in PBS with 4% paraformaldehyde and 10% sucrose before being embedded in OCT compound and frozen. Five micrometer thick sections of the frozen spleens were obtained on a microtome and fixed in chilled acetone for 10 min followed by washes in PBS-Tween (0.5 %). For immunostaining, all incubations were performed in a humidified chamber at RT. Sections were blocked with 2.4G2 (Biolegend, CA) and 5% rat serum for 20 min. The following antibodies were used for staining: anti–IgD-biotin followed by Streptavidin-Alexa Fluor 555 (1:400; invitrogen), anti-mouse–IgM-APC (1:200; Biolegend) and anti-mouse-MOMA-1 (1:300; Serotec) or anti-mouse CD209b (Clone# 2C7B27, Biolegend). Sections were washed extensively in PBS-Tween followed by PBS (10 mM) and dehydrated by sequential washes with 95 and 100% ethanol. After drying for 15–20 min, coverslips were mounted with anti-fade mounting medium (Invitrogen).

### Reconstitution of the lymphoid compartment in Rag-1–deficient mice

Rag-1^−^/^−^ mice were irradiated with 700 rads and reconstituted with 5 × 10^6^ WT or mutant adult BM cells via tail vein injection. Reconstitution of the BM and spleen was assessed 7 wk later by flow cytometry.

### Cell number calculation and statistical analysis

Cell numbers were calculated by enumerating lymphocyte yields from each primary/secondary lymphoid organ analyzed using a hemocytometer. Absolute counts of each subset analyzed were calculated by normalizing the data based on proportion of each subset within the lymphocyte gate with the total cell yield from the respective lymphoid organ.

Significance was calculated by means of the Student 2-tailed *t*-test assuming 2 samples with unequal variance. Results for which *P* < 0.05 were considered statistically significant.

## Author contributions

FA, HM, DZ, AC, DV, and HH performed all the studies. HM, HH, JA, and SP planned the studies and wrote the manuscript.

### Conflict of interest statement

The authors declare that the research was conducted in the absence of any commercial or financial relationships that could be construed as a potential conflict of interest.
