# Supplementary material for: The Mst1 Kinase Is Required for Follicular B Cell Homing and B-1 B Cell Development
Source: Front Immunol. 2018 Oct 17;9:2393. doi: 10.3389/fimmu.2018.02393 (PMC6199389; doi:10.3389/fimmu.2018.02393)

**
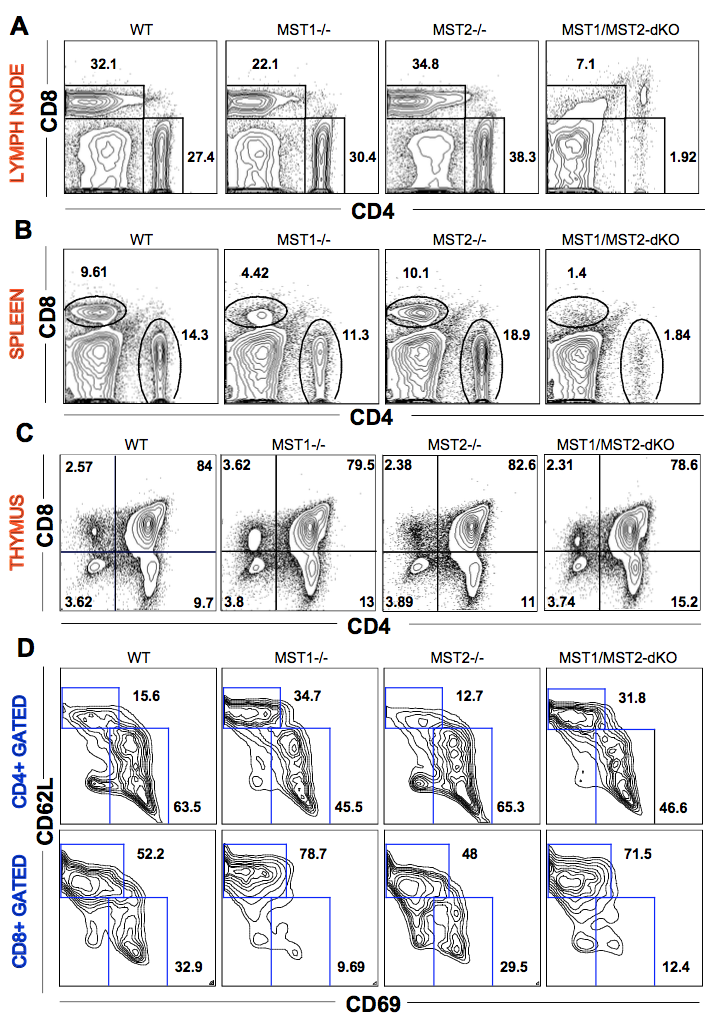
Supplementary figure 1: Flow cytometry analysis of lymphocytes from primary and secondary lymphoid organs of Mst1-/- and Mst1/Mst2-dKO mice.** (Data are representative of 6 mice analyzed from all groups)

**Supplementary figure 2: Lymphopenia in Mst1/Mst2-dKO mice.**

Flow cytometric analysis show a reduction of CD4+ and CD8+ T cells and IgD+ B cells in peripheral blood in Mst1/2-dKO compare to WT control littermates. (Data are representative of peripheral blood analyzed from 5 WT and 10 Mst1/Mst2-dKO mice)

**
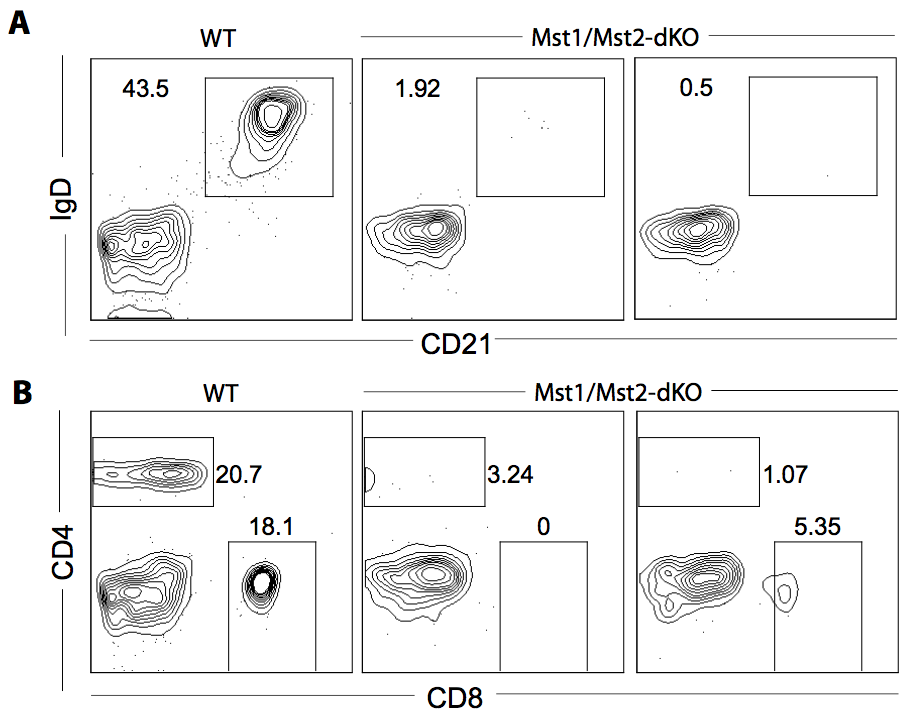
**

**Supplementary figure 3**: **Reduced BCR signaling in B cell subsets from Mst1-/- and Mst1/Mst2-dKO mice.** Top panel shows the gating strategy for different B cell subsets analyzed. The lower panels show the ability of each sub-fraction to flux intracellular Calcium. Results are representative of two independent experiments.


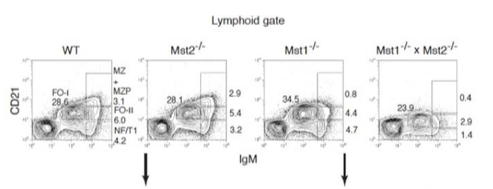

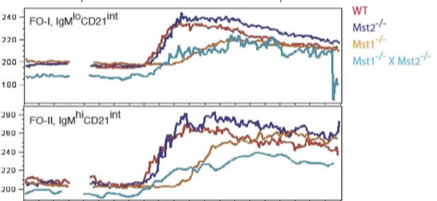

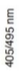

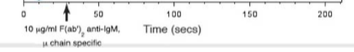

Supplement: Supplementary file 1 [file Data_Sheet_1.docx]
